# Models of Supervision Relevant To Peer or Lived Experience Workforce Within Mental Health: A Scoping Review

**DOI:** 10.1007/s10597-025-01581-7

**Published:** 2026-01-16

**Authors:** Liam Hodge, Tania Pearce, Bess Jackson, Sarah Wayland

**Affiliations:** 1 University of New England, Armidale, Australia; 2 Central Queensland University, Rockhampton, Australia

**Keywords:** Peer work, Supervision, Clinical framework, Peer work supervision

## Abstract

This scoping review explores the alignment between clinical supervision models and the values underpinning Peer Work within the mental health sector. Peer Work, grounded in lived experience and principles of empowerment, mutuality, and recovery, has become an integral component of mental health services in Australia and internationally. Despite its growing presence, Peer Work lacks a defined supervision framework tailored to its unique ethos. Using the PRISMA-ScR methodology and Arksey and O’Malley’s five-stage framework, 2,434 records were screened, resulting in 23 studies that met the inclusion criteria. These studies were analysed against the six foundational pillars of Peer Work as defined by Mind Australia. Findings indicate that while several clinical supervision models, such as the Seven-Eyed Model, DBT-informed supervision, and strengths-based approaches demonstrate theoretical alignment with Peer Work values, practical implementation remains limited. Recovery orientation and empowerment were the most commonly aligned themes, while trauma-informed care and inclusivity were less frequently addressed. The review highlights a critical gap in supervision tailored to Peer Workers and calls for further qualitative research and co-designed frameworks to ensure supervision practices uphold the integrity of Peer Work. Without such development, there is a risk of diluting Peer Work’s transformative potential within traditional clinical systems.

## Introduction

### Background

The Peer Workforce has become known for providing mutual support and driving advocacy and systemic change (Meagher & Naughtin, 2018). The First Australian National Mental Health Plan (1993–98) marked the formal inclusion of consumer advocates as stakeholders in service delivery. Consequently, consumer participation in decision-making and care rose from 33% in 1993 to 68% in 2003, reflecting increased Peer Work engagement (Rosen, 2006). In 2014, there were an estimated 40 full-time equivalent (FTE) Peer Workers in Australia’s mental health sector. By 2020/2021, the national average rose to 103.8 FTE Peer Workers per 10,000 mental health staff, with New South Wales reporting a higher rate of 137.5 (Byrne et al., 2021). The National Mental Health Commission recognises Peer Work and Lived Experience work as a distinct discipline within the mental health system (Byrne et al., 2021).

Not only in Australia, the global interest in employing Peer Workers to support mental health recovery is growing. In other countries, the UK and the USA, Peer Workers are increasingly recognised as a complementary component of mental health service delivery. Drawing on their lived experience, they are uniquely positioned to offer hope, support, and connection to others navigating the mental health system (Byrne et al., 2021). Recent Australian Government funding trends and workforce data suggest a significant expansion of the Peer Workforce in the coming years (Butler, 2025; Scanlan et al., 2020).

In an effort to support the ongoing growth of the workforce, the National Lived Experience (Peer) Workforce Development Guidelines were introduced, approximately 70 years after peer support services first began in Australia (Byrne et al., 2021). These guidelines marked the initial effort to clarify the role of Peer Workers within the mental health sector, the report does not describe or operationalise a framework or model for supervision practice for peer workers. Prior to this, Peer Workers were often overlooked and disconnected from the broader system, facing numerous challenges. They are expected to deliver psychosocial support and act as advocates for systemic change, despite lacking formal recognition and workforce support (Seal et al., 2024; Wyder et al., 2020). According to Rothwell et al. (2021), supervision constitutes a fundamental component of safe professional practice. It not only facilitates the development of professional competencies, through skill enhancement and knowledge transfer, supervision offers a reflective and supportive environment, crucial for mitigating burnout and sustaining long-term engagement in practice.

Peer Worker supervision is not offered or is haphazard, and its delivery is via line management, increasing the risk of harm to the workforce when not applied from the co-designed evidence base (Seal et al., 2024; Wyder et al., 2020). Within the mental health sector, it is important to challenge the narrative, that Peer Work involves employing individuals who are ‘lesser’ or less skilled, simply to reserve more complex tasks for professionals (Meagher & Naughtin, 2018). Currently, there is no formal accreditation process or established model of supervision for Peer Workers. Although supervision is widely recognised as a core component of practice in health and mental health professions, such opportunities remain limited in Peer Work (Castles et al., 2023). Peer Workers practise within a defined framework, shaped by their specific roles and responsibilities.

This paper distinguishes itself from the work of Castles et al. (2023) by building upon the existing literature in the field. While Castles et al. (2023) focused on establishing Peer Work supervision practices and operationalising supervision within workplace settings, this review offers a critical analysis of the space between Peer Work values and current supervision practices in mental health. Therefore, establishing a base of evidence for models that may provide support to Peer Workers in the mental health field. Through a theoretical lens this paper aligns current supervision practices alongside the six pillars of Peer Work. Therefore, embedding supervision practice that acknowledges the values held by Peer Work workforce.

### Understanding the Scope of Peer Work Inclusion

For this review, the Authors have utilised the Mind Australia Peer Work Framework, to define the scope of the Peer Workforce and the foundations that underpin it (Childs, 2021). The purpose for this choice was Mind’s position in Australia as a leading peak for Peer Work integration and their evidence-based framework that has been developed in collaboration with Peer Workers and people with a Lived Experience of mental distress.

Peer Workers contribute to a wide range of programs and are involved across multiple systems within the mental health sector. In Australia, for example, Peer Work is embedded in areas such as mental health, alcohol and other drugs (AOD), and homelessness services. Their roles often include advocacy, consultancy, education and training, academic collaboration, and peer-led support initiatives (Victorian Department of Health, 2023). Peer Work has significantly enriched community mental health by strengthening psychosocial support and introducing innovative approaches to service delivery for individuals with mental health needs (Fong et al., 2018).

Anti-oppressive practice, activism, and advocacy, which originated from the social work movement, have strong links to Peer Work practice. Similar to critical social work frameworks, Peer Work aims to empower users, provide non-judgmental spaces for care, and offer freedom of choice (Meagher & Naughtin, 2018). Social work is based on principles of social justice and human rights, advocating for change, empowerment, and the liberation of people (International Federation of Social Workers, 2025). The concept of being *advocates for change* within a system is not new and has been integral to social work theory and practice since its inception, especially for social workers in mental health settings (Bland et al., 2021). However, without adequate supervision, support, and training, much like in social work, this can lead to burnout and a significant reduction in capacity (Lived Experience Australia, 2019; Meagher & Naughtin, 2018).

There is a strong regard for the Peer Workforce in Australia, but there are also significant barriers that the Peer Workforce experience (Hughes et al., 2017). Many of these challenges include low expectations from workplaces, stigma, segregation, and disconnect from the service (Meagher & Naughtin, 2018). One of the ways to address the experiences of working in any role in the mental health sector is through access to evidenced based supervision.

### Current Peer Workforce Supervision Models

Current supervision models for Peer Workers lack published or publicly available evidence to shape engagement. The structures and support mechanisms that aim to sustain Peer Work have not kept pace with the growth of the workforce, raising concerns that the Peer Workforce may be reintegrated into the traditional mental health system rather than being seen as their own workforce with specific workplace needs (Sinclair, 2018). The Peer Workforce has consistently resisted the biomedical model within the mental health system. Without evidenced-based supervision, there is a significant risk that Peer Workers could lose the radical aspect, that initially brought them into the sector and be subsumed into the clinical workforce.

Supervision is widely recognised in healthcare settings as a fundamental component of evidence-based practice. Research indicates that clinical supervision supports both the development and sustainability of the psychological profession (Falender, 2018). Similarly, social workers and other mental health professionals have established supervision structures that support their registration with professional bodies. Supervision typically provides three core functions: administrative oversight, educational guidance, and emotional or practical support (Kadushin and Harkness, 2002, in Castles et al., 2023). Its effectiveness lies in offering professional support that benefits both practitioners and clients. Supervision fosters reflective practice, reduces burnout, and improves staff retention, thereby enhancing the work environment and client outcomes (Martin et al., 2021). These positive outcomes are what we aim to replicate within the Peer Work context.

Literature exploring the development of the Lived Experience and Peer Workforce has identified the need for discipline-specific supervision to uphold the integrity of Peer Work, support role clarity and provide a reflective space for personal and professional growth (Byrne et al., 2021). However, the evidence for implementation is minimal. Peer Workers are consistently and routinely inserted into multidisciplinary teams, often under the routine supervision of clinicians (Castles et al., 2023). Clinical supervision without designated, discipline-specific supervision places Peer Workers at risk of unintentional harm, operating in ambiguity (Castles et al., 2023). Understanding current models of supervision used in Peer Work, both clinical and discipline-specific, and their effectiveness, is vital to the safe expansion of the Peer Workforce.

### Aims/Objective

This scoping review sought to explore the current clinical supervision practice and frameworks available for mental health professionals, to map these frameworks against the values and concepts of Peer Work. Through analysis of clinical supervision models compared alongside the values/frameworks relevant to Peer Work, the goal is to identify which models align.

## Terms

Table 1 outlines key terms and their definitions for the purpose of this scoping review.

**Table 1:** Key terms and definitions

Term	Description
Peer Work	A broad definition of Peer Work is applied capturing all those who work in the mental health sector, but these terms are still contested. However, regardless of position or knowledge, this manuscript acknowledges that all persons have lived experience, and those who bring this experience to their work each day carry a different load. Peer Work has its basis in mutuality and respect (Meagher & Naughtin, 2018) for one another and the shared experience. This is unlike a clinician or support worker, who may have a similar experience but does not carry the need to disclose their experience. It is this disclosure that makes Peer Work a special and difficult place to operate. As a sign of respect for people who have earned the right, this document will use the capitalised version of Peer Work throughout. Acknowledging that each of these people brings with them a story of recovery and hope for the future.
Mental Health System	For this paper, the mental health system is a space where people can seek support from professionals, be that clinical staff or Peer Workers. For this instance, clinical staff will be defined as those who work under a registration (Mental Health Social Worker, Psychologist, psychiatrist, etc.) and paraprofessionals and are utilising psychological-based therapies for the support of a client. This could be in a hospital setting such as a community mental health program, or it could relate to a walk-in support program.
Supervision	Although it remains a contested term across many sectors of mental health and can have different meanings across different professions. In this instance, this manuscript will use the following definition: *Structured supervision is focused primarily on four functions: professional growth in the practice area; critical analysis; professional and personal support; and accountability in the provision of services*,* management*,* and administration (*Australian Association of Social Workers, 2023*).*

## Methodology

Munn et al. (2018) highlighted that the purpose or aim of a scoping review is to provide an overall description of theories and concepts where there is limited information. The exploratory nature of the scoping review allows for the gathering and synthesis of a broad range of knowledge. This broad range of knowledge capture allows an informed understanding of supervision practice and the links to Peer Worker practice. The scoping review was conducted using the Systematic Reviews and Meta-analysis Protocols Extension for Scoping Reviews Guidelines (PRISMA-ScR) (Shamseer et al., 2015). The research followed the Arksey and O’Malley (2005) framework and its extensions (Colquhoun et al., 2014).

The five steps set forth by Arksey and O’Malley (2005) to conduct the scoping review as set out below:

### Identifying the Research Question

Supervision is a critical aspect of clinical practice, what is not known is the impact and role of supervision on the Peer Worker experience. To understand this, a review of evidence-based frameworks that may support Peer Worker discipline specific supervision is required. A search of literature and an understanding of gaps in knowledge, led the team of co-authors to conceptualise the following question: *What theories and models of supervision align with the values of Peer Work within the mental health sector?*

The authors published a protocol using the PRISMA-ScR guidelines. Published on Research Gate alongside updates for inclusion and exclusion criteria. The protocol was published on 25 April 2024 (Hodge et al., 2024).

### Identification of Studies

A suitable team, with content and methodological expertise, was established early in the process to ensure a successful completion of the review. The search string was developed and tested in collaboration with the Author 2, an information specialist, to ensure accurate results and capture variations in the mental health workforce and Peer Worker terms.

The initial stage of research involved the searching of several academic databases for peer-reviewed; Scopus, EBSCO (including CINHAL), PubMed, and ProQuest database searches were conducted on the 1 st of September 2024. An example of a search string used to search ProQuest appeared as: ab, ti(Peer N/3 Work* OR Lived N/1 Experience N/3 Work* OR Peer N/3 Support* OR Peer N/3 Counsel* OR Peer N/3 Mentor*) AND ab, ti(Model OR Approach OR Framework) AND ab, ti(Supervis* OR Individual N/3 supervision OR Group N/1 Supervision OR Peer N/3 Supervision OR Role Supervisor OR Manager OR Mentor* OR Coach*) AND ab, ti (“mental health” OR “mental healthcare” OR health and well N/1 being).

Given the evolving nature of the field due to increased interest from governments and agencies, it was important to include publications produced outside peer-reviewed sources. Keyword searches were conducted across both Google and Google Scholar (Searches conducted on 01/09/2024) utilising the “site: gov.au” and “site: org.au” modifiers. The results were then imported into EndNote V.20, then exported to Covidence for screening and review. Searches were not restricted by year but were restricted by language (English only), no other parameters were placed on the search.

This paper extended the search strategy employed by Castles et al. (2023) with the development of a structured Boolean search across Scopus, EBSCO (including CINAHL), PubMed, and ProQuest. Additionally, the 2024 strategy expanded its grey literature search using Google and Google Scholar with domain-specific modifiers and employed EndNote and Covidence for systematic screening, whereas the Castles et al. (2023) review relied on manual searches and expert consultations. These differences reflect a progression toward greater precision, breadth, and methodological rigor in this paper’s approach. Finally, the theoretical application of the Peer Work pillars to the supervision models provides a clear distinction between the two papers.

### Inclusion/exclusion Criteria

Screeners were provided with a scope of the inclusion and exclusion criteria, along with an information session, facilitated by Author 1. The session was designed to provide them with adequate resourcing to complete the screening process. The session allowed screeners time to ask questions and complete a mock screening to examine challenges that could arise throughout the process. This induction step kept conflicts to a minimum and ensured all screeners were seeking the same information. Screeners were provided with a decision tree to support decision-making and clarity through the screening process, providing them with a framework when operating in isolation. This process allowed the screening to take place with rigour and ensure a consistent approach.

Eligibility criteria included English language only, clinical roles in mental health including Peer Workers, Mental Health Clinicians, Social Workers, Psychologists, Psychiatrists, Mental Health Nurses, and Nurses, in addition to an explicit definition of a supervision framework utilised and a rationale for its inclusion.

Exclusion criteria included: Protocol papers, Peer workforces in sectors outside of mental health including Alcohol and Other Drugs (AOD), HIV, or other settings.

No time period was set given the relative infancy of the sector.

### Screening

Authors 1 and 3, along with an additional independent reviewer (a University of New England Higher Degree Research student), screened the records by both title and abstract. Conflicts were managed by an independent third party, Author 2. Results were then passed to the screeners for full-text review. Author 2 completed a blind review of the texts that were in conflict, the final texts were either accepted or removed from the study.

Some conflicts arose due to the nature of the wording and terms within mental health. There was some confusion regarding settings and the population study, for example inclusion or exclusion regarding the training of clinical students and PhD students in a clinical setting was captured through ‘supervision’. The term ‘supervision’ was too broad and included unwanted information.

A total of 2,434 records were imported into Covidence for screening. After title and abstract screening, 2,179 records were excluded for not meeting the eligibility criteria (e.g., editorials, conceptual papers, duplicates). The remaining 255 articles underwent full-text review, of which 232 were excluded due to reasons such as unavailability of the full text, irrelevant participants, inappropriate study design or publication type, or lack of a model or framework. Ultimately, 23 studies met the inclusion criteria and were included in the final review (Table 2) (Page et al., 2021).

**Table 2:**
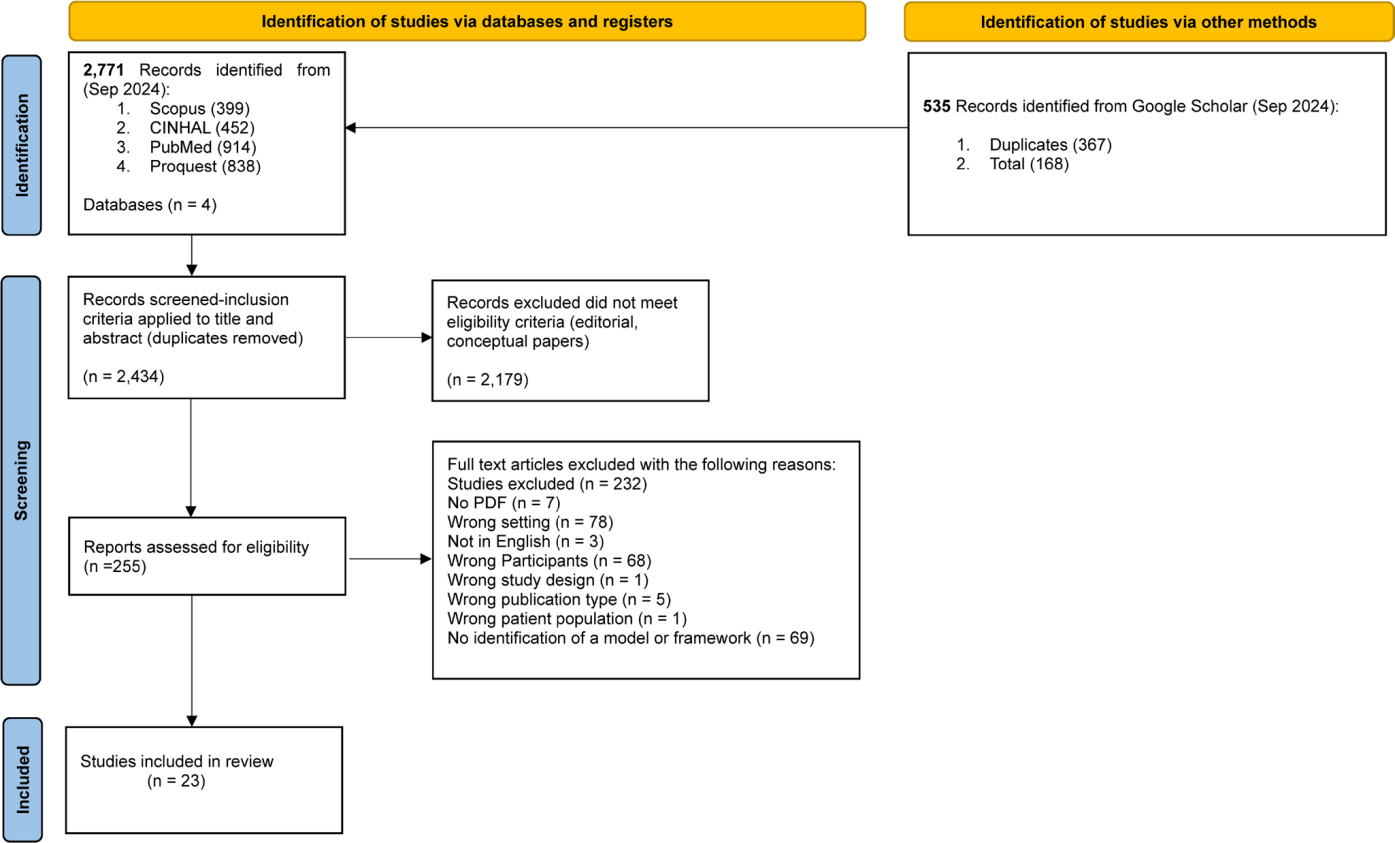
PRISMA statement

### Charting the Data

After the screening process, records were then linked with the different key themes of Peer Work as outlined by Mind Australia (Childs, 2021). Mind is a Peer Worker and Lived Experience led non-government organisation who drive research and innovation across Peer Work in Australia. Their framework outlines the core competencies of Peer Work and the values that uphold Lived Experience practice and are defined in Table 3.

**Table 3:** Peer work pillars

Peer Work Pillars	Description	Supervisory Competency
Hope/Recovery orientated practice	To maintain a positive and optimistic outlook; valuing hope, courage and perseverance; knowing that people do recover from mental health challenges. Using Lived Experience of recovery, holding onto hope for someone else ad incorporate their firsthand perspectives.	The supervisory model reflects language that is supportive of hope and recovery for clients and practitioners. The emphasis is on supporting the client through to recovery and not simply treatment for symptoms.
Strengths Based Practice/Advocacy	Peer Workers have a unique skill to build relationships that both acknowledge and minimise the power imbalance, to build connection based on trust.	Supervisory methods focus on leveraging the inherent strengths and competencies of nursing supervisees. Supervisors facilitate reflective analyses that empower students to identify and amplify their strengths, subsequently enhancing their ability to address client needs effectively.
Trauma-Informed Practice	By drawing on Lived Experience, Peer Workers understand the trauma on some people’s lives and take this into their practice.	The importance of understanding the impact of trauma on individuals. Significant importance on creating a safe space for open discussions, a foundational element of trauma-informed care. By fostering understanding of trauma’s impact and avoiding re-traumatisation.
Mutuality and Reciprocity	Building relationships which minimise power imbalances and build connection and trust; empathising – the ability to take the perspective of another and feel the way they feel. Being aware of body language, developing and maintaining connection, avoiding problem solving or giving advice, being curious, listening and learn and reflecting language back to the person. Avoiding clinical or diagnostic terms.	The supervision process involves collaborative goal-setting and discussion of expectations, fostering mutual learning and support between supervisors and supervisees. This mirrors mutuality and reciprocity in peer support.
Inclusivity and Respect	Considering the rights, values, beliefs and property of all people. Acting with honesty and accountability. Sharing personal Lived Experience in a way that is both safe and appropriate. Reducing stigma and validating other’s experience, inspire hope and demonstrate empathy.	Awareness of cultural and cohort-specific factors is highlighted as essential for promoting inclusivity. Supervisors are encouraged to create environments where diverse perspectives and experiences are valued and respected.
Empowerment and Self Determination.	Making a difference - Working towards social justice, respect for people’s rights and fostering the inclusion of consumers in community life. Knowing that understanding experiences, behaviours and beliefs is key to the process of recovery.	The model promotes empowerment by encouraging supervisors to facilitate supervisees’ exploration of their thoughts, values, and beliefs. Supporting and valuing the Lived Experience of the supervisee. This approach fosters self-determination and supports individuals in taking control of their lives.
	(Childs, 2021)	

Papers were searched using a content analysis framework (Pollock et al., 2023). Each paper was assessed for alignment with the core research themes. This included identifying the presence of specific keywords, concepts, or theoretical constructs central to the study. Key findings were summarised and categorised to identify patterns, contradictions, or gaps in the literature. Contributions to theory, practice, or policy were also noted. The key frameworks were placed in a table (Table 4) allowing the Authors to view the themes of each paper and align them with Peer Work practice. Supervisory Competency, in this instance, was the frameworks demonstrated ability to provide supervision that is congruent with peer work philosophy, actively reinforce peer work pillars in practice, and creates conditions where peer workers could develop their unique strengths while maintaining the integrity of peer work principles. This process was done by content analysis of the models, understanding the information which underpins the model and mapping this into a table in an Excel spreadsheet. From this data analysis authors were then able to represent the models as a table and score each article. Papers were scored on the number of references to themes of Peer Work foundations of practice. Papers were allocated a score out of six, whereby, they met all the criteria of alignment with Peer Work. Scores and frameworks are discussed below, Charting was completed by Author 1. Author 1 then presented the content analysis to Co-authors gaining agreement across all domains and main themes of the content.

**Table 4:** Framework alignment

Author (Year)	Framework	Framework Insights	Participants	Peer Work Foundations					
				1	2	3	4	5	6
Abassary and Goodrich (2014)	CARE Model of Supervision	• Contextualised supervision • Action response and focus • Empathetic supervisory relationship	Counsellors	Y	Y	Y	Y	Y	Y
Andersson et al. (2010)	Mindfulness Based Role Play Supervision	• Experiential recovery‑focused learning • Empowering reflective practice • Inclusive mutual support	Mental Health Practitioners	Y	N	N	N	Y	Y
Bodley (1992)	Process Record of Supervision	• Creative recovery-oriented supervision. • Reflective strengths-based practice. • Flexible trauma-informed support.	Psychiatric Nurses	Y	Y	Y	Y	Y	Y
Bussey and Jemal (2023)	Anti-Racist Mental Health Supervision	• Action‑oriented recovery practice • Empowering strengths‑based agency • Inclusive mutual sanctuary	Mental Health Practitioners	Y	Y	Y	Y	Y	Y
Coll et al. (2024)	Bernard’s Discrimination Model	• Structured recovery‑focused supervision • Empowering strengths‑based growth • Inclusive collaborative practice	Training Clinicians	N	Y	N	Y	Y	N
Dallimore et al. (2016)	Multidisciplinary team (MDT) clinical supervision	• Collaborative recovery-oriented dialogue • Inclusive strengths-based supervision • Safe trauma-informed reflection	Mental Health Practitioners	Y	Y	Y	Y	Y	Y
Darongkamas et al. (2014)	Hawkins and Shohet’s clinical supervision model	• Empowering focused development • Relational development • Collaborative inclusive supervision.	Counsellors	N	N	N	Y	Y	Y
Emelianchik-Key et al. (2022)	Dialectical Behaviour Therapy Skills into Supervision	• Recovery‑focused skill development • Emotionally safe supervision • Empowering collaborative practice	Clinicians	Y	Y	Y	Y	Y	Y
Feinstein (2021)	Cognitive Apprenticeship Model	• Reflective recovery-oriented learning • Empowering strengths-based growth • Collaborative inclusive supervision.	Training Clinicians	Y	Y	N	N	Y	Y
Gutierrez et al. (2020)	Spiritually Competent Orientation	• Spiritually informed recovery orientation. • Collaborative trauma‑aware supervision. • Inclusive strengths‑based practice.	Mental Health Practitioners	N	Y	Y	Y	Y	Y
Guindon et al. (2022)	Cognitive Behavioural Supervision	• Collaborative recovery‑focused supervision. • Empowering structured learning. • Mutual inclusive practice.	Training Clinicians	Y	N	N	Y	N	N
Knight (2018)	Trauma Informed Model of Supervision	• Trust-based trauma-informed supervision. • Empowering strengths-focused practice. • Collaborative inclusive recovery.	Mental Health Practitioners	Y	Y	Y	Y	Y	Y
Legha (2023)	Antiracist Approach to Mental Health Supervision	• Transformative recovery‑focused practice • Empowering antiracist supervision • Collaborative inclusive growth	Mental Health Practitioners	Y	Y	Y	Y	Y	Y
McMahon et al. (2022)	Seven-Eyed Model of Supervision	• Empowering recovery‑focused development. • Strengths‑based relational practice. • Inclusive mutual supervision.	Mental Health Practitioners	Y	Y	N	Y	N	N
McMahon et al. (2023)	Herons Six Category Intervention Framework	• Supportive recovery‑focused supervision • Strengths‑based empowering practice • Inclusive mutual learning	Training Clinicians	Y	N	Y	Y	Y	Y
Merizzi (2019)	Seven Eyed Model Vs Cyclical Model	• Relational recovery-oriented supervision • Strengths-based inclusive practice • Reflective trauma-informed support.	Psychologists	Y	N	Y	Y	Y	Y
Nelson et al. (2015)	Cultural and Dual Supervision	• Developmental recovery-oriented growth • Flexible strengths-based supervision • Inclusive empowering practice	Aboriginal and/or Torres Strait Islander counsellors	Y	Y	Y	Y	Y	Y
Nelson et al. (2000)	Blended Integration Approach to Supervision	• Culturally safe supervision models • Inclusive strengths-based learning • Remote mutual recovery support	Mental Health Practitioners	N	N	N	Y	Y	Y
Pullman et al. (2023)	Strengths Based Supervision	• Recovery‑oriented strengths practice • Empowering collaborative supervision • Inclusive client‑centred growth.	Mental Health Practitioners	Y	Y	Y	Y	N	Y
Sewell and Ederer (2023)	SNAP (Stop Now And Plan) Supervision	• Competency‑focused recovery support • Empowering structured development • Inclusive collaborative supervision.	Mental Health Practitioners	N	Y	N	N	N	N
Soliman (2023)	Circle of Security and Supervision	• Relational recovery‑focused support • Strengths‑based reflective practice • Empowering mutual growth	Mental Health Practitioners	Y	N	N	Y	N	Y
Son et al. (2022)	Attachment and Supervision	• Supportive recovery‑focused alliance • Empowering inclusive supervision • Mutual respectful growth	Mental Health Practitioners	Y	Y	N	N	N	N
Triantafillou (2011)	Solution Focused Supervision	• Solution Orientated Supervision • Flexible and worker focused • Structured implementation	Mental Health Practitioners	Y	Y	N	N	N	N

1 = Hope/Recovery Orientation, 2 = Strengths-Based Practice, 3 = Trauma-Informed Care, 4 = Empowerment and Self-Determination, 5 = Mutuality and Reciprocity, 6 = Inclusivity and Respect

### Collating and Reporting

A total of 23 articles, that met the inclusion criteria in terms of demonstrating a supervision model in the mental health sector were located. Each paper outlined a unique supervision model tailored to address the specific needs of a particular cohort. *Mental Health Practitioners* was used as a catch all term to shorten a group when more than two different practitioners were used in the study, this occurred in 50% of the articles. The next largest group of participants was Training Clinicians (Including training psychologists or other training clinical staff), since to maintain registration or complete registration this cohort is mandated to attend supervision.

11 studies used qualitative methods, interviewing supervisors and supervisees about their experiences with specific supervision models. Eight studies conducted literature reviews, mainly focusing on supervision for mental health practitioners. Three were individual case studies, and one used a cross-cultural comparative design.

The studies were published between 1992 and 2023. Of these, eight (35%) emphasised the importance of all six core Peer Work values in their supervision models. Most studies showed strong alignment with Peer Work principles, particularly Recovery and Empowerment, which were highlighted in 78% of the research. Strengths and Mutuality were each noted in 70% of studies, while Inclusivity appeared in 74%. Only 52% addressed Trauma-Informed Supervision or related practices.

### Consultation

Author 1 identifies as having a Lived Experience of mental illness and suicidality. Author 1 has experience in working in roles impacted by the lack of supervision in mental health programs. Other authors have learnt experience working on programs with Peer Workers and have offered expertise and advice surrounding the complexity of the system. This paper is designed to understand the current evidence based supervision models in the mental health sector, forming the basis for future collaboration with the Peer Work sector, where genuine co-creation can take place (Pearce et al., 2022).

## Types of Models Identified

Overall, there were 23 different models identified across the articles, these were then mapped against the 6 pillars of Peer Work, outlined by Childs (2021). There were no models of supervision that focused on people with a Lived Experience, all models were from a clinical perspective.

### Recovery Orientated Practice

Four studies placed greater emphasis on Recovery Orientation than on other Peer Work principles (Andersson et al., 2010; Bodley, 1992; Dallimore et al., 2016; Triantafillou, 2011). Firstly, Bodley (1992) proposed a structured, reflective supervision model for psychiatric nursing students, centred on process recording and reflective practice. This approach supports client autonomy and aligns with recovery-oriented care.

Building on this foundation, Andersson et al. (2010) introduced a Mindfulness-Based Role-Play model, which is more experiential than traditional supervision. It enhances empathy and self-awareness through techniques like the “two-chair intervention,” encouraging mutual learning in a safe, reflective environment.

Enhancing the structured models of supervision, Dallimore et al. (2016) developed a model based on clinical discussion groups within multidisciplinary teams. This model promotes collaboration and recovery-focused care, even in acute settings, by fostering open dialogue and psychological input.

The supervision model proposed by Triantafillou (2011) presents a solution-focused, client-centred supervision model. It helps supervisees align their practice with client goals, promoting empowerment, self-determination, and professional accountability.

### Inclusivity and Respect

Only one model was aligned to this pillar (Nelson et al., 2015), however this pillar theoretically overlaps with the pillar of mutuality and reciprocity, thus other models may partially align. Nelson et al. (2015) outline supervision models for supporting Aboriginal and Torres Strait Islander mental health workers, focusing on effectiveness and accessibility, especially in remote areas. Dual supervision models, involving clinical and cultural supervisors, address both dimensions, enhancing supervision quality. Practitioners emphasised creating inclusive environments that value diversity and respect all individuals. Honouring diverse experiences is essential for effective mental health support. These approaches highlight the importance of culturally informed, person-centred supervision in advancing mental health care (Nelson et al., 2015).

### Trauma-Informed Care/Empathy

Two studies placed Trauma-Informed Practice at the centre of their supervision models, emphasising trust and understanding (Gutierrez et al., 2020; Knight, 2018). Knight (2018) integrated Trauma-Informed Practice into Bernard’s Discrimination Model (Bernard & Goodyear, 1998), enhancing supervisors’ ability to support those working with trauma survivors. The model prioritises trust, safety, empowerment, and ongoing dialogue, helping supervisees avoid common errors and better support clients. Empowerment is key, to promoting strategies that foster autonomy and emotional regulation.

Gutierrez et al. (2020) combined spiritual and cultural frameworks with supervision, incorporating the Multicultural Competent Orientation, spiritual competencies from the Association for Spiritual, Ethical, and Religious Values in Counselling, and the Cultural Third approach. This model creates a safe, collaborative space that prevents re-traumatisation and supports healing. While focused on spiritually competent supervision, it also aligns with core Peer Work values such as recovery orientation, strengths-based practice, empowerment, mutuality, and inclusivity.

### Strengths Based Practice

Five studies aligned with strengths-based and recovery-oriented supervision practices in the mental health sector (Feinstein, 2021; Legha, 2023; Nelson et al., 2000; Pullman et al., 2023; Sewell & Ederer, 2023). Nelson et al. (2000) proposed an integrated model combining developmental and discrimination frameworks, offering flexible, strengths-focused supervision that supports supervisee growth. Similarly, Feinstein’s (2021) Cognitive Apprenticeship Model emphasises experiential learning through modelling, feedback, and reflection, building confidence and resilience, principles consistent with recovery-oriented practice.

Providing a reflexive and clinical framework for children’s services, Sewell and Ederer (2023) introduced the SNAP Supervision model. SNAP provides supervision for children’s mental health services, focusing on goal setting, feedback, and accountability. While not directly transferable to Peer Work, its emphasis on practitioner development and competence reflects strengths-based values.

In the Australian context, Pullman et al. (2023) implemented a Strengths Model in public mental health services. Their approach integrates individual and group supervision, field mentoring, and structured training, promoting a hopeful, client-centred practice that strengthens therapeutic relationships.

Adding a critical and systemic dimension, Legha (2023) presented the Anti-Racist Mental Health Supervision model, grounded in Critical Race Theory and inspired by Kendi’s How to Be an Antiracist (2019). This model challenges hierarchical structures, promotes institutional change, and fosters collaborative, reflective supervision. It includes six action steps aimed at dismantling systemic racism and empowering both supervisors and trainees.

### Empowerment and Self-determination

Five studies focused on empowerment and self-determination, linking these concepts to strengths-based supervision (Abassary & Goodrich, 2014; Darongkamas et al., 2014; Emelianchik-Key et al., 2022; McMahon et al., 2022, 2023). Emelianchik-Key et al. (2022) integrated Dialectical Behaviour Therapy (DBT) skills into supervision to reduce stress and enhance supervisees’ communication and decision-making. This approach supports self-determined professional growth.

Complementing this, McMahon et al. (2022) explored the Seven-Eyed Model of Supervision (Hawkins & McMahon, 2020), which focuses on relational dynamics and professional development. It supports empowerment and mutual learning through reflective practice. Furthering this relational focus, McMahon et al. (2023) applied Heron’s Six Category Intervention Framework (Heron, 2001), which distinguishes between directive and facilitative styles. The model encourages supportive, autonomy-promoting supervision and is recommended for supervisor training.

A more crisis-based response, Abassary and Goodrich (2014) introduced the CARE Model for crisis and trauma supervision. It includes four components, context, action, response, and empathy, providing structured support and promoting critical thinking and self-determination during crises.

Finally, Darongkamas et al. (2014) expanded Hawkins and Shohet’s model (Hawkins & Shohet, 2012) by adding an eighth mode, ‘observing us’, inspired by Cognitive Analytic Therapy. This reflexive, collaborative approach enhances mutual understanding and empowers supervisees through shared reflection.

### Mutuality and Reciprocity

Six studies demonstrated a strong alignment with the principle of mutuality, highlighting the central role of the supervisory relationship in achieving positive outcomes in clinical supervision (Bussey & Jemal, 2023; Coll et al., 2024; Guindon et al., 2022; Merizzi, 2019; Soliman, 2023; Son et al., 2022). Merizzi (2019) examined the application of the Seven-Eyed Model and the Cyclical Model within older adult mental health services. By integrating these frameworks, the model offers a comprehensive, multidimensional approach that addresses the complex needs of supervisees. The process fosters mutual learning and encourages critical reflection on age-related biases and stereotypes, reinforcing the values of reciprocity and shared growth.

Developing a collaborative model, Guindon et al. (2022) introduced the Tandem Model of supervision, using the metaphor of a tandem bicycle to illustrate the collaborative journey between supervisor and supervisee. While the supervisor provides direction, both parties contribute to progress toward shared goals. The model emphasises the importance of creating a psychologically safe environment, reducing power imbalances, and promoting active participation in the learning process. Similarly, Son et al. (2022) explored the supervisory working alliance in both South Korean and U.S. contexts, noting its significance in fostering supervisee openness and reducing nondisclosure. The study highlighted the importance of cultural sensitivity and regular check-ins to maintain trust and mutual engagement, which are essential for empowerment and self-determination.

Coll (2024) applied Bernard’s Discrimination Model, focusing on four key areas: professional behaviour, process skills, conceptualisation, and personalisation. The model supports supervisee development through structured feedback and reflective dialogue. The collaborative consultant role within the model encourages shared responsibility for learning and promotes mutual growth.

The Critical Transformative Potential Development model (Bussey & Jemal, 2023) integrates anti-racist principles into supervision. It focuses on five key areas: affinity, awareness, accountability, agency, and action. The model fosters a “supervisory sanctuary” where supervisors and supervisees engage in collective reflection and action, promoting inclusivity, empowerment, and mutual support.

Using the Circle of Security framework, Soliman (2023) developed a relational supervision model grounded in attachment theory. The model includes six components that address relational dynamics, attachment needs, and reflective practice. It emphasises mutual influence and shared vulnerability, creating a space for collaborative learning and professional development.

### Main Domains that Emphasise Peer Work

The majority of models reviewed (78%) emphasised the Peer Work pillars of Recovery and Empowerment, with those incorporating Trauma-Informed Practice and Inclusivity aligning most closely with all Peer Work criteria. In contrast, models lacking a Trauma-Informed foundation often fail to address essential elements such as Mutuality and Inclusiveness, an expected outcome given that Trauma-Informed Practice is grounded in principles of safety, trust, choice, collaboration, and empowerment (NSW Health, 2022). These findings suggest that a Trauma-Informed Practice framework is fundamental to effectively understanding and implementing Peer Work supervision.

## Discussion

This review sought to understand the literature exploring clinical supervision models to better understand their alignment or misalignment with Peer Work Pillars, defined by Mind Australia. The history of Peer Work is embedded in social justice and championing for change in the face of an established system by those who have experienced using the services within the system. Evidence has demonstrated across many health fields, including mental health that employees from all disciplines benefit from adequate support and supervision; decreasing the risk of vicarious trauma and increasing the longevity of mental healthcare workers in the workplace (Falender, 2018; Yirtici, 2025). Peer Workers, who continue to operate in the mental health space and are now an established and relied-upon workforce, should be offered the same support structure as their colleagues (Meagher & Naughtin, 2018). This should align with support that is consistent with Peer Work values and seeks to support Peer Work’s core mission which is empowerment and self-determination of service in the mental health system (Meagher & Naughtin, 2018).

The findings from this review indicates there are several approaches that may better suit the Peer Work workforce. Some clinically driven models of supervision seamlessly fit alongside Peer Work Practice and frameworks from a theoretical perspective (Abassary, 2014; Bodley, 1992; Bussey, 2023; Dallimore et al., 2016; Emelianchik-Key et al., 2022; Knight, 2018; Legha, 2023). Despite their clinical focus, models such as DBT skills in supervision (Emelianchik-Key et al., 2022), Process Record (Bodley, 1992), and multi-disciplinary team supervision (Dallimore et al., 2016) models all aligned with the key pillars in Peer Work. Each of these models scored highly on the rating scale utilised in this review, covering six areas associated with Peer Work practice. The Process Record highlighted key aspects of recovery-oriented practice, reflective supervision and flexibility, recognising the dynamic nature of the workforce the framework supports (Bodley, 1992). The DBT skills in supervision model provides a focus on strengths-based practice and trauma-informed care, highlighting the importance of a supportive supervisory relationship (Emelianchik-Key et al., 2022). Finally, the Multi-disciplinary team supervision highlights the power of recovery-oriented practice alongside empowerment and self-determination, encouraging staff to take responsibility for their learning; this empowerment translates to improved patient support, enabling individuals to make informed choices regarding their recovery and well-being (Dallimore et al., 2016). Although none of the models is grounded in trauma informed practice, each underscores its critical importance, a key cornerstone of Peer Work practice, and thus show promise to contribute toward peer work specific supervision framework development.

However, despite these theoretical alignments, significant tensions and constraints exist that challenge the integration of clinical supervision models into Peer Work. There is a tension between Peer Work and current mental health practices (Byrne et al., 2021), which may hinder the integration of clinically driven models into Peer Work supervision. Models grounded in clinical practice or focused on outcomes and managerialism would struggle to be implemented in Peer Work given the origins of the workforce. Further, the extant literature highlights that supervisors may not understand the unique role and value of Peer Workers, which in turn may lead to inadequate support and guidance (Meagher & Naughtin, 2018). There are strong positive links between highly clinical models of supervision and the pillars of Peer Work, yet evidence highlights that Peer Workers in clinical settings can feel undervalued and stigmatised (Meagher & Naughtin, 2018). A well-designed supervision model must acknowledge that Peer Workers can experience stigma and discrimination from other staff, which may undermine their morale and effectiveness. This can also occur between Peer Workers themselves, especially when roles are unclear, undefined, or poorly integrated, leading to conflict and negatively impacting wellbeing (Janoušková et al., 2022; Ser et al., 2024). Ambiguity around the boundaries and responsibilities of Peer Workers is common, particularly within multidisciplinary teams, and can further complicate their role (Meagher & Naughtin, 2018). The evidence base demonstrates a disconnect between the clinical workforce and the Peer Workforce, pointing to high rates of discrimination towards Peers within the sector (Meagher & Naughtin, 2018).

Given the inconsistent nature of Peer Work supervision approaches and the predominance of clinically oriented line management, uncertainty remains about how best to deliver peer work in mental health settings. In the absence of standardised qualification pathways or accreditation processes for peer workers, supervision may be overlooked in favour of workload or managerial support. While there are strong theoretical links between clinical supervision models and the pillars of Peer Work, it remains unclear whether these models would align with Peer Work in practice, particularly given the significant constraints evident in clinical supervision itself. The extant literature identifies that many clinical supervisees do not receive adequate time or funding to complete supervision (Rothwell et al., 2021), raising critical questions about how discipline-specific supervision can be integrated into the Peer Work sector without adequate evidence, funding, and support. Currently, several constraints impact clinical supervision that must be addressed in the Peer Work context, including time limitations, organisational support needs, ethical challenges, the duality of relationships, and training and education gaps (Rothwell et al., 2021).

It is the evidence of this disconnect, the understanding of these theoretical models, and the recognition of systemic constraints that highlights the need for a collaborative, co-created model of supervision, developed with mutuality and empowerment. The models identified in this review can be positioned along a spectrum of theoretical suitability for Peer Work supervision yet require input from those working in peer worker roles to consider suitability, feasibility and practicality. Doing so offers a pathway toward a co-created supervision framework with Peer Workers, providing a foundation to understand how best to provide supervision to the workforce developed to support this growing mental health workforce. Given the nature of the Peer Work sector and the Pillars which underpin the discipline, co-creation in a space of mutuality is critical to the development of a new framework, as co-creation of knowledge is essential in developing new and emerging areas in health, improving outcomes, and fostering collaboration (Pearce et al., 2022). Pearce et al. (2022) highlight that co-creation represents a shared relational process of generating understanding and solutions with, rather than, for others. It builds research capacity through collaboration and respectful engagement where all voices hold value, including practitioners, service users, and community members. Given the nature of Peer Work and its underpinning values of respect and mutuality, this review provides an evidence base for the co-creation of a Peer Work supervision framework that honours these principles and addresses the unique supervisory needs of the sector. This review has highlighted some foundations which can be utilised to commence this co-creation process to develop and test a peer work specific supervision model.

## Limitations

This paper acknowledges an overreliance on the theoretical alignment of clinical supervision models within Peer Work. While it presents a foundation of evidence for models that may inform and enhance Peer Work supervision, there remains significant scope for advancement through empirical research. Future studies should prioritise consultation with Peer Workers and employ qualitative methodologies to explore the relational dynamics of supervision and the practical application of these models in Peer Work contexts.

Although this review evaluates models based on their alignment with Peer Work values, it does not offer empirical evidence that these models have been tested, adapted, or evaluated specifically within Peer Work settings. This limitation affects the external validity of the findings and raises concerns about the transferability of these models to Peer Work environments. Further research is needed to assess their relevance and impact in practice. Additionally, the study was constrained by the evolving and inconsistent terminology used within Peer Work and the broader mental health sector. Despite employing a comprehensive and well-developed search strategy, some relevant terms or phrases may not have been captured, potentially limiting the scope of the review.

## Conclusion

The evidence has shown that there are models of supervision developed within the clinical mental health sector that would align with Peer Work and Lived Experience practice. Models embodying the principles of Peer Work, including, recovery orientation, empowerment, trauma informed practice were revealed. What is not clear from this review is the practical implications of these models within the Peer Work sector. Some of the overtly clinical models, while aligned in theory, are highly structured, clinician-centric, and may not accommodate the flexibility, autonomy, and relational dynamics that are central to Peer Work. This contradiction highlights the gap between theoretical compatibility and practical usability. It is therefore important that further study of the Peer Work sector is undertaken, and a qualitative study is performed to understand what is currently being delivered. Without critical interrogation of how these models might reshape or dilute Peer Work values in practice, the application of supervision models utilised in clinical settings, without further consultation, risks reinforcing the very power dynamics Peer Work seeks to challenge.

## Data Availability

No datasets were generated or analysed during the current study.
